# Effects of photobiomodulation on oral mucositis, oral pain, xerostomia, salivary flow rate, and quality of life in patients with head and neck cancer: a systematic review and meta-analysis

**DOI:** 10.1007/s00520-026-10575-4

**Published:** 2026-03-25

**Authors:** Mônica N. M. Pedroso, Anderson Rech, Natalia Casagrande, Júlia F. Pissaia, Julia S. Zardo, Vitória L. Borges, Stephanie R. Bombana, Cindranne Torres Muller, Priscila Casara, Janaína Brollo, Claudio P. Júnior, Carin W. Gallon, Pedro Lopez

**Affiliations:** 1 Programa de Pós-Graduação em Ciências da Saúde, Universidade de Caxias do Sul, Caxias do Sul, Rio Grande do Sul Brazil; 2 Grupo de Pesquisa Em Exercício Para Populações Clínicas (GPCLIN), Universidade de Caxias do Sul, Caxias do Sul, Rio Grande do Sul Brazil; 3 Curso de Educação Física, Universidade de Caxias do Sul, Caxias do Sul, Rio Grande do Sul Brazil; 4 Curso de Medicina, Universidade de Caxias do Sul, Caxias do Sul, Rio Grande do Sul Brazil; 5 Hospital Geral de Caxias do Sul, Caxias do Sul, Rio Grande do Sul Brazil; 6 Curso de Nutrição, Universidade de Caxias do Sul, Caxias do Sul, Rio Grande do Sul Brazil; 7 Sociedade Brasileira de Nutrição Oncológica (SBNO), Rio de Janeiro, Rio de Janeiro Brazil; 8 School of Medical and Health Sciences, Edith Cowan University, Joondalup, WA Australia; 9 Pleural Medicine Unit, Institute for Respiratory Health, Perth, WA Australia

**Keywords:** Head and neck neoplasms, Low-level light therapy, Mouth dryness, Quality of life

## Abstract

**Purpose:**

To systematically review and analyze the effects of photobiomodulation on oral mucositis, xerostomia, salivary flow rates, oral pain, and quality of life in patients with head and neck cancer.

**Methods:**

A systematic search of six databases was conducted up to April 2025. Eligible randomized controlled trials (RCTs) examined the effects of photobiomodulation on the specified outcomes in patients with head and neck cancer. Pooled estimates (risk ratio [RR] and standardized mean difference [SMD]) were calculated using a three-level mixed-effects meta-analysis.

**Results:**

Thirty-three articles describing 30 RCTs (*n* = 1748) were included. Photobiomodulation significantly reduced the risk of severe oral mucositis (RR 0.46, 95% CI: 0.29–0.71, *P* < 0.001) and severe oral pain (RR 0.35, 95% CI: 0.23–0.53, *P* < 0.001) compared with controls. Significant improvements were also observed for salivary flow rate (SMD 0.75, 95% CI: 0.03–1.46, *P* = 0.044). No significant effects were observed on xerostomia (SMD −0.07, 95% CI: −0.47–0.33, *P* = 0.646) and quality of life (SMD 1.06, 95% CI: −0.03–2.14, *P* = 0.060). The benefits were consistent across different photobiomodulation protocols for oral mucositis and quality of life.

**Conclusions:**

Photobiomodulation appears to be a promising supportive care intervention, reducing the risk of severe mucositis and oral pain, while potentially improving salivary flow rates and preserving quality of life in patients with head and neck cancer. However, the certainty of the evidence ranged from very low to low and most included studies presented a high risk of bias; therefore, these findings should be interpreted with caution.

**Supplementary Information:**

The online version contains supplementary material available at 10.1007/s00520-026-10575-4.

## Introduction

Head and neck cancer is the third most prevalent cancer worldwide, accounting for ~ 800,000 incident cases and 400,000 deaths annually [9]. Curative treatment for these malignancies often includes radiotherapy (50 to 70 Gy) [76] combined with cisplatin-based chemotherapy [54, 64], resulting in improved disease-free and overall survival [7, 14]. However, a range of acute and chronic side effects are associated with these therapies, including oral mucositis [73] and xerostomia/hyposalivation [5], affecting from 60 up to 90% of patients undergoing primary treatment. The onset of these complications often leads to treatment interruptions, enteral nutritional therapy (feeding tube) dependence [34], and prolonged hospitalisation [34], ultimately affecting treatment efficacy and survival as well as diminishing quality of life during treatment.

Photobiomodulation therapy has emerged as a promising noninvasive intervention for mitigating cancer treatment-related toxicities [33, 77]. Experimental evidence indicates that photobiomodulation can promote increased mitochondrial activity and adenosine triphosphate (ATP) synthesis and upregulates growth factors and anti-inflammatory mediators [17, 22], thereby potentially accelerating mucosal healing and restoring salivary gland function [80, 81]. Based on these underlying mechanisms and accumulated clinical data [40, 61, 67], photobiomodulation has been recommended by the *Multinational Association of Supportive Care in Cancer and the International Society of Oral Oncology* (MASCC/ISOO) [21] and *World Association of photobiomoduLation Therapy* (WALT) [69] for the prevention of severe oral mucositis in patients receiving radiotherapy, with or without concomitant chemotherapy. However, these recommendations [21, 69] were primarily derived from studies undertaken in different cancer groups, which limits the precision and applicability of photobiomodulation protocols specifically to patients with head and neck cancer, despite the similarity of symptoms induced by anticancer treatments.

Previous systematic reviews investigating the effects of photobiomodulation in patients with head and neck cancer [10, 19, 65, 71] were mostly limited to qualitative synthesis, small number of studies, and outcomes. Among the few meta-analyses available, Campos et al. [10] reported that photobiomodulation reduced the risk of severe oral mucositis by 64% after analysing six trials. Similarly, Shen et al. [71] found beneficial effects from the second week of treatment onwards, although with variations in the magnitude of benefits across studies. Additionally, most of the available systematic review evidence has focused on oral mucositis, limiting the understanding of undertaking photobiomodulation to improve the treatment experience and outcomes of patients with head and neck cancer. Therefore, a contemporary and comprehensive quantitative synthesis of the photobiomodulation literature is warranted to evaluate the effects of photobiomodulation on oral mucositis, oral pain, xerostomia, salivary flow rates, and quality of life in this population.

As a result, this systematic review and meta-analysis aimed to evaluate the effects of photobiomodulation on oral mucositis, xerostomia, salivary flow rates, oral pain, and quality of life in patients with head and neck cancer. Such information can guide the development of standardized clinical protocols and support evidence-based recommendations for an optimal integration of photobiomodulation into multidisciplinary cancer care.

## Methods

All methodological procedures in this study followed the guideline of the Cochrane Back Review Group (CBRG) [25] and adhered to the Preferred Reporting Items for Systematic Reviews and Meta-Analyses (PRISMA) statement [42, 60]. The protocol for this systematic review was prospectively registered in the International Prospective Register of Systematic Reviews (PROSPERO) under the registration number CRD420251014400.

### Eligibility criteria

Studies were included in this systematic review if meeting the following criteria regarding participants, intervention, comparator, outcomes and study design (PICOS): (i) studies involving adult patients diagnosed with head and neck cancer who had received or receiving primary cancer treatment (radiotherapy and/or chemotherapy); (ii) studies involving photobiomodulation protocols; (iii) studies implementing a non-exposed control group, such as those with no formal intervention, mouthwash, or placebo as comparators; (iv) studies evaluating mucositis, xerostomia, salivary flow rate, oral pain, and quality of life using validated questionnaires, protocols or scales; and (v) randomized controlled trials. The exclusion criteria were as follows: (i) studies involving adult patients diagnosed with head and neck cancer who had preexisting oral mucositis and (ii) studies published in a language other than English, Portuguese, or Spanish.

### Search strategy and study selection procedure

A comprehensive literature search was conducted by one researcher (PL) across six databases: *CINAHL*, *Embase*, *LILACS*, *PubMed*, *Scielo*, and *Web of Science*, from inception to 21 April 2025, with an updated search in PubMed performed on 26 February 2026. *Embase* was added after protocol development to improve search coverage. In addition, the Cochrane Library was searched to identify ongoing trials over the past five years. The search strategy used controlled vocabulary and free-text terms, as detailed in the Supplementary Appendix S1. To complement the exhaustive search, a manual review of references list from included studies (i.e., backward citation chasing) was also performed to identify additional eligible articles. During the screening phase, titles and abstracts were first independently evaluated following the eligibility criteria. Eligibility was assessed independently in duplicate (JFP, JSZ, and NC), with differences resolved by a third reviewer (PL) when disagreements occurred. In case of abstracts that did not provide sufficient information, they were selected for full-text evaluation. Full-text articles meeting criteria were retrieved and read independently by both reviewers and assessed for inclusion in the study.

### Data extraction

Data extraction was performed by multiple researchers (JFP, JSZ, NC, SRB, and VLB) and checked by another researcher (PL) via a standardized form. Study information including sample size, age, type of cancer, number of male participants, cancer stage, metastasis, and radiotherapy and/or chemotherapy regimen. In addition, we extracted information regarding the photobiomodulation protocol, including duration, weekly and daily frequency, number of sessions, light source, emission spectrum, emission mode, average power output, and beam spot size at the target area. Further details about outcomes extraction are presented in the Supplementary Material.

For binary data, the incidence of severe mucositis and oral pain were assessed for the longest period of the intervention. Continuous outcomes were assessed at baseline and for the longest period of the intervention. Both within- and between-group mean difference extracted in their absolute units and for the longest period of the intervention. For studies that did not report dispersion measures for the change scores (e.g., standard deviation [SD], standard error, or 95% confidence intervals), SDs of the change were imputed assuming a correlation coefficient of r = 0.5 between baseline and post-intervention measures, using the square root of $((SD_{Baseline}^{2} + SD_{Post-intervention}^{2})-(2 \times r \times SD_{Baseline}\times SD_{Post-intervention}))$  [39]. Additional analyses were performed using a correlation of *r* = 0.25 and 0.75 (Supplementary Material**)**. When outcome data were available only in graphical form, numerical values were extracted using WebPlotDigitizer (San Francisco, CA) [20]. Further information on study risk of bias assessment and certainty of evidence (GRADE) is presented in the Supplementary Material.

### Data analysis

A robust variance estimation approach was undertaken to account for the nested structure of the effect sizes calculated from the studies included (i.e., effects nested within categories nested within studies) [12]. This approach was undertaken for mucositis, xerostomia, salivary flow rate, oral pain levels, and quality of life given the availability of several dependent outcomes from the same study. Therefore, a three-level mixed-effects meta-analysis with *study* included as a random effect was performed to examine the effect of the photobiomodulation protocols on the outcomes of interest.

For dichotomous outcomes such as severe oral mucositis and oral pain, a meta-analysis of risk ratios (RR) was conducted. Risk ratios and their 95% confidence intervals were computed using log-transformation and pooled using a random-effects model. The results were then back transformed and reported as RRs to facilitate clinical interpretation. The pooled effect estimated from the continuous outcomes were obtained and expressed as SMD. Cluster robust point estimates 95% confidence intervals (95% CI) were provided, weighted by inverse sampling variance to account for the within- and between-study variance (*τ*^2^). Restricted maximal-likelihood estimation was used in all models. Statistical significance was assumed when the SMD was below an *α* level of *P* ≤ 0.05. According to Cohen [13], SMD values of 0.0 to ≤ 0.5 represent small; 0.51 to 0.79, medium; and ≥ 0.8, large effects.

Statistical heterogeneity was assessed using the Cochran *Q* test. A threshold *P* value of 0.1 and values greater than 50% in *I*^2^ were considered indicative of high heterogeneity. Publication bias was explored by contour-enhanced funnel plots and Egger’s test [63] when more than 10 studies were available. Subgroup analyses were provided for (i) photobiomodulation protocols and (ii) assessment method when more than 10 studies were available, with robust estimates produced for each subgroup, and fixed effects with moderator’s model were used to compare the models. Analyses were conducted using the package *metafor* [75] and *clubSandwich* [66] from R (R Core Team, version 4.0.3., 2020).

## Results

A total of 1312 records were identified through the systematic search. After removing duplicates, 1007 records were screened based on title and abstract. Of these, 566 records were excluded as irrelevant to the research question, resulting in 441 records eligible for full-text review. A total of 411 reports met the exclusion criteria, while three additional studies [6, 46, 74] was identified through reference lists. Finally, 33 articles [2, 4, 6, 15, 16, 18, 23, 26–30, 37, 41, 43, 45–52, 55–59, 68, 70, 74, 79, 82] describing 30 randomized controlled trials (*n* = 1748 patients) were included in this systematic review.

Data from 28 studies [2, 4, 6, 15, 16, 18, 23, 26–29, 37, 43, 45–47, 49–52, 55–57, 59, 68, 70, 74, 82] were further included in the meta-analyses. The selection process is presented in Supplementary Figure S1, whereas study, participant, and intervention characteristics and risk of bias are detailed in the Supplementary Material. Regarding ongoing or recently registered trials, a total of 11 studies were identified over the past five years (RBR-4hbxk9k; CTRI/2024/08/072614; RBR-10v5gcdk; NCT05614843; IRCT20080906001216N3; ISRCTN14224600; NCT03972527; RBR-5h4y4n; NCT05106608; RBR-9bt3cbj; RBR-5746z9).

### Oral mucositis

Twelve effects across 11 studies were included in the model for oral mucositis. A total of 274 cases of severe mucositis were reported among 707 patients [2, 4, 18, 26–29, 37, 43, 49–51, 55, 59], corresponding to a cumulative incidence of 38.8%. Severe mucositis was assessed using different grading systems (WHO, RTOG, and NCI), which are broadly comparable in identifying clinically significant mucositis but differ in thresholds and clinical interpretation. Subgroup analysis by mucositis grade assessment indicated statistically significant differences (*χ*^2^ = 7.8, *P* = 0.020). Studies using the WHO scale (RR 0.24, 95% CI: 0.05 to 1.07, *P* = 0.206) and RTOG scale (RR 0.34, 95% CI: 0.22 to 0.53, *P* = 0.020) displayed greater reductions in mucositis relative risk compared with those using the NCI staging (RR 0.65, 95% CI: 0.32 to 1.29, *P* = 0.150).

**Fig. 1:**
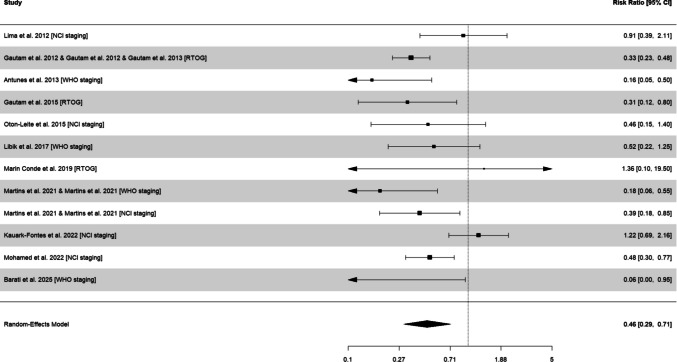
Random-effects meta-analysis of the effect of photobiomodulation on the risk of severe oral mucositis in patients with head and neck cancer. 95% CI, 95% confidence interval

The overall analysis indicated that photobiomodulation significantly reduced the risk of severe oral mucositis compared with controls, with a pooled risk ratio (RR) of 0.46 (95% CI: 0.29 to 0.71, *P* < 0.001), which represents a 54% relative risk reduction in severe mucositis in the photobiomodulation group (Table 1 and Fig. 1). This pooled estimate represents an overall approximation of treatment effect across related but not fully equivalent definitions of severe mucositis. The heterogeneity *I*^2^ was 57%, with no presence of publication bias (*P* = 0.596; Supplementary Figure S7). A sensitivity analysis omitting an outlier [37] resulted in a slightly greater effect of photobiomodulation (RR 0.39, 95% CI: 0.28 to 0.54, *P* < 0.001), and a reduction in heterogeneity *I*^2^ to 6%. The certainty of evidence was deemed low.

**Table 1:** Photobiomodulation effects on the incidence of severe mucositis and severe pain in patients with head and neck cancer

Outcomes			Random effect meta-analysis			Heterogeneity			Certainty of evidence
	*k*	*n*	RR	95% CI	*P*-value	*Q*	*I*^*2*^	*P*-value	
Severe mucositis	11	12	0.46	0.29 to 0.71	< 0.001	26.5	57%	0.005	⊕ ⊕ ⊝ ⊝ Low ^a,b^
Severe pain	3	3	0.35	0.23 to 0.53	< 0.001	4.6	42%	0.208	⊕ ⊕ ⊝ ⊝ Low ^a,c^

95% CI, 95% confidence intervals, *n* number of effect sizes, *I*^2^ percentage of variation across studies that is due to heterogeneity, *k* number of studies, *Q* Cochran’s *Q* test of heterogeneity

^a^Certainty of evidence downgraded due to most studies (> 50%) presenting with high risk in the risk of bias assessment

^b^Certainty of evidence downgraded due to inconsistency, with heterogeneity above 50%

^c^Certainty of evidence downgraded due to the small number of studies and lack of publication bias assessment

Subgroup analysis by light source (*χ*^2^ = 1.3, *P* = 0.250) and emission spectrum (*χ*^2^ = 0.0, *P* = 0.924) indicated no differences. Studies using diode (RR 0.49, 95% CI: 0.25 to 0.98, *P* = 0.047) or He–Ne (RR 0.35, 95% CI: 0.17 to 0.71, *P* = 0.030) derived significant benefits. Comparable effects were also observed for protocols of 600–660 nm (RR 0.46, 95% CI: 0.26 to 0.80, *P* = 0.013) and 810–940 nm (RR 0.47, 95% CI: 0.12 to 1.08, *P* = 0.091), although the latter did not reach statistical significance.

### Oral pain

Three effects across three studies [2, 26, 28] were included in the RR model for oral pain. One study [4] reported no cases of severe oral pain using the *Oral Mucositis Weekly Questionnaire* and was not included for further analysis. A total of 90 cases of severe oral pain (≥ 7 in visual analogue scale [VAS]) were reported among 250 patients, corresponding to a cumulative incidence of 36.0%. Photobiomodulation significantly reduced the risk of severe oral pain compared with controls, with a pooled RR of 0.35 (95% CI: 0.23 to 0.53, *P* < 0.001). This represents a 65% relative risk reduction in severe oral pain favouring the photobiomodulation group (Table 1). The heterogeneity *I*^2^ was 42%. The certainty of evidence was deemed very low.

Six effects across six studies [2, 6, 15, 45, 55, 74] were included in the continuous model for oral pain, using VAS and the pain domain of *EORTC QLQ C-30*. There were no significant differences between photobiomodulation and control groups for oral pain levels, despite the large SMD of −2.34 (95% CI: −5.54 to 0.85, *P* = 0.151). The overall heterogeneity *I*^2^ was 98%.

### Xerostomia and salivary rate

Six effects across five studies [43, 46, 47, 70, 74] were included in the model for xerostomia using a visual analogue scale (VAS), the *Treatment-Emergent Symptom Scale* and the *Xerostomia Inventory*. There were no significant differences between photobiomodulation and control groups for xerostomia, with a SMD of −0.07 (95% CI: −0.47 to 0.33, *P* = 0.646; Table 2). The heterogeneity *I*^2^ was 0%. The certainty of evidence was deemed very low.

**Table 2:** Photobiomodulation effects on oral pain, xerostomia, salivary flow, and quality of life in patients with head and neck cancer

Outcomes			Random effect meta-analysis			Heterogeneity			Certainty of evidence
	*k*	*n*	SMD	95% CI	*P*-value	*Q*	*I*^*2*^	*P*-value	
Oral pain	6	6	−2.34	−5.54 to 0.85	0.151	99.1	98%	< 0.001	⊕ ⊝ ⊝ ⊝ Very low ^a,b,c,d^
Xerostomia	5	6	−0.07	−0.47 to 0.33	0.646	1.3	0%	0.931	⊕ ⊝ ⊝ ⊝ Very low ^a,c,d^
Salivary flow	6	10	0.75	0.03 to 1.46	0.044	12.5	37%	0.188	⊕ ⊝ ⊝ ⊝ Very low ^a,c,d^
Quality of life	11	27	1.06	−0.03 to 2.14	0.060	145.2	88%	< 0.001	⊕ ⊝ ⊝ ⊝ Very low ^a,b,c,d^

95% CI, 95% confidence intervals, *n* number of effect sizes, *I*^2^ percentage of variation across studies that is due to heterogeneity, *k* number of studies, *Q* Cochran’s *Q* test of heterogeneity

^a^Certainty of evidence downgraded due to most studies (> 50%) presenting with high risk in the risk of bias assessment

^b^Certainty of evidence downgraded due to inconsistency, with heterogeneity above 50%

^c^Certainty of evidence downgraded due to the small number of studies and lack of publication bias assessment, or evidence of publication bias

^d^Certainty of evidence downgraded due to imprecision, with confidence intervals from interventions crossing null values or including values favouring both interventions tested

Ten effects across six studies [43, 45–47, 56, 74] were included in the model for salivary rate flow. Photobiomodulation resulted in a significant moderate effect compared with controls, with an overall SMD of 0.75 (95% CI: 0.03 to 1.46, *P* = 0.044) based on both stimulated and unstimulated salivary flow (Table 2). This effect was consistent across both stimulated (0.75 SMD, 95% CI: −0.32 to 1.82, *P* = 0.110) and unstimulated salivary rate (0.69 SMD, 95% CI: −0.16 to 1.54, *P* = 0.090), although they did not reach statistical significance. The overall heterogeneity *I*^2^ was 37%. The certainty of evidence was deemed very low.

### Quality of life

**Fig. 2:**
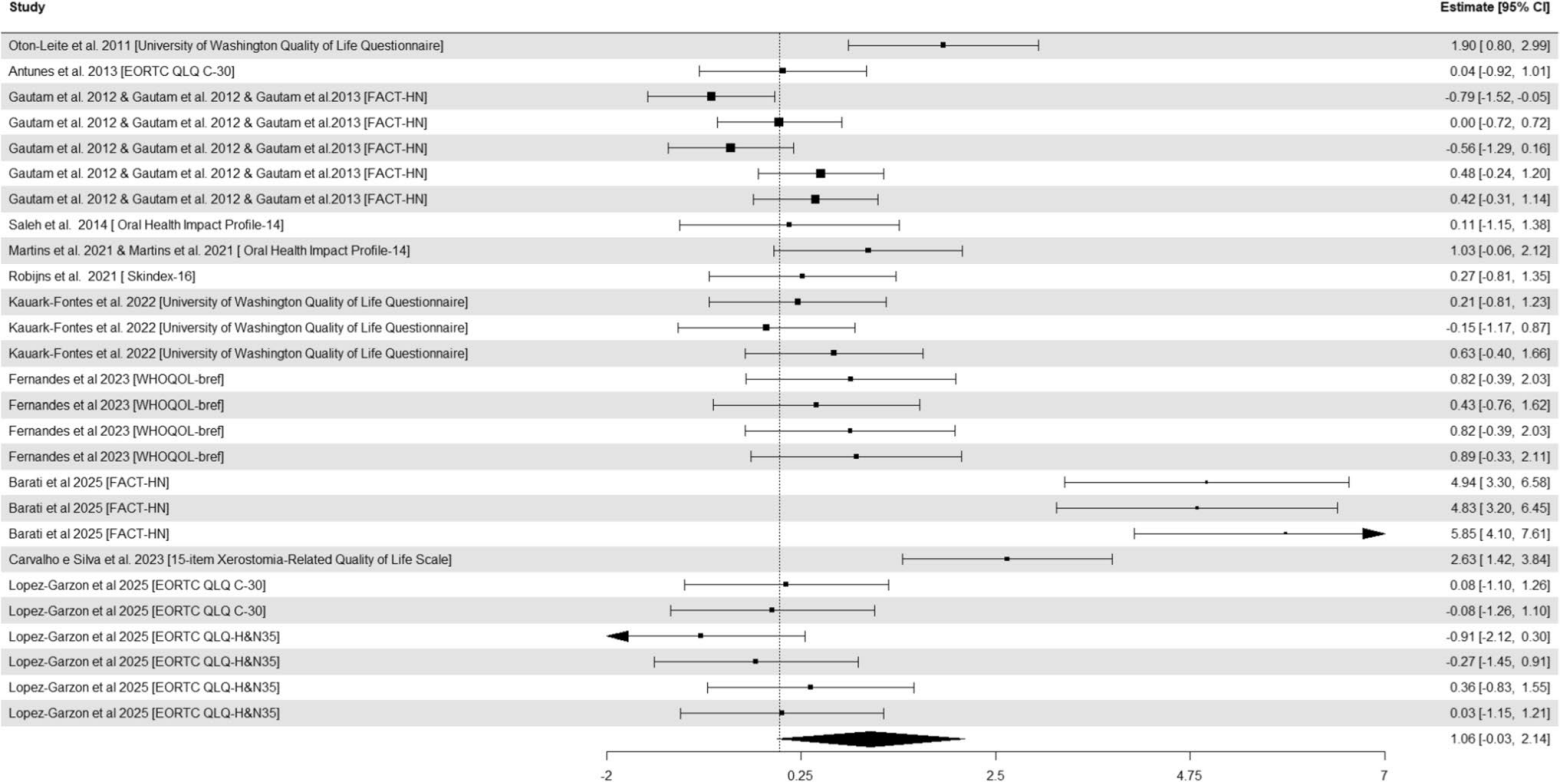
Random-effects meta-analysis of the effect of photobiomodulation on the quality of life in patients with head and neck cancer. 95% CI, 95% confidence interval

Twenty-seven effects across 14 studies [2, 4, 16, 23, 27–29, 37, 46, 50, 51, 57, 68, 70] were included in the model for quality of life using a variety of validated instruments, including the *University of Washington Quality of Life Questionnaire*, the *Functional Assessment of Cancer Treatment-Head and Neck*, the *EORTC QLQ C-30*, the *Oral Health Impact Profile-14*, the *Skindex-16*, the *WHOQOL-bref*, and the *15-item Xerostomia-Related Quality of Life Scale*. Photobiomodulation resulted in a significant large SMD of 1.06 (95% CI: −0.03 to 2.14, *P* = 0.060; Fig. 2 and Table 2) compared with controls. The overall heterogeneity *I*^2^ was 88%. Evidence of publication bias was detected (*P* < 0.001; Supplementary Figure S8), and studies were not added after applying the trim-and-fill method. The certainty of evidence was deemed very low.

Subgroup analysis by emission spectrum indicated no differences (*χ*^2^ = 1.2, *P* = 0.272). Studies using protocols of 808–940 nm (1.46 SMD, 95% CI: −0.72 to 3.63, *P* = 0.146) displayed greater effects on quality of life than those using 600–660 nm (0.46 SMD, 95% CI: −0.50 to 1.41, *P* = 0.243), although they did not reach statistical significance. Subgroup analysis by light source were not undertaken given the lack of sufficient studies using He–Ne protocols (*n* = 1).

## Discussion

This study aimed to systematically review and analyze the effects of photobiomodulation on oral mucositis, xerostomia, salivary flow rates, oral pain, and quality of life in adults with head and neck cancer. We found that photobiomodulation was associated with a 54% reduction in the risk of severe mucositis and a 65% reduction in the risk of severe oral pain, despite the low certainty of evidence. Although potential increases in salivary flow rates were observed, no statistically significant effects were found for xerostomia. These benefits were accompanied by large but uncertain improvements in quality of life. Despite the low certainty of evidence, our findings support the potential role of photobiomodulation in attenuating treatment-related toxicities and preserving quality of life in adults with head and neck cancer.

According to the most recent clinical practice guidelines [21, 69], photobiomodulation is recommended for the prevention of oral mucositis in patients with head and neck cancer undergoing radiotherapy, with or without concomitant chemotherapy. In our analysis, we observed a potential preventive effect, with photobiomodulation reducing the risk of severe mucositis by 54%, which is in line with a recent meta-analysis [71]. A range of nutritional aspects, including dietary intake, nutritional status, appetite loss, and unintentional weight loss [11, 31], are affected by the development of oral mucositis, resulting in a cycle where mucositis and related symptoms such as oral pain, chewing and swallowing difficulties exacerbate one another. Our results suggest that reductions in severe mucositis may translate into lower risks of debilitating acute reactions, feeding tube placement [34], infections [72], hospitalization [34], and ultimately, mortality [61]. In addition, these were observed compared with usual care, commonly including mouthwash and basic oral care used in clinical practice. Collectively, these potential effects may contribute to improved treatment tolerance and adherence and fewer treatment interruptions, although this remains to be confirmed in future studies.

Nevertheless, the implementation of photobiomodulation for oral mucositis remains challenging in clinical practice due to the lack of protocol standardisation, including treatment frequency, dose, emission spectrum and anatomical application sites. For example, we found that less than 50% of the studies used emission spectrum between 630 and 660 nm, which is the recommended by MASCC/ISOO [21] and WALT [69], while the remaining studies presented substantial variation in the protocols, reaching up to 940 nm using intraoral or transcutaneous device [49]. We found a comparable reduction in the risk of severe mucositis with the use of higher emission spectrum (810 to 940 nm), although this finding was based on a small number of studies included and did not reach statistical significance. It remains uncertain whether higher wavelengths confer additional therapeutic benefits or, conversely, whether they may pose potential risks that outweigh the benefits already achieved with the recommended protocols [21]. Regarding light source, diode-based photobiomodulation is deemed the gold standard, and its specific use was associated with relevant reductions in the risk of severe mucositis, like those achieved with He–Ne. Based on the available evidence, diode-based photobiomodulation with emission spectrum ranging from 632.8 to 660 nm should remain the preferred approach, whereas higher emission spectrum may be reserved for patients with anatomical limitations or when targeting deeper tissues and salivary glands in transcutaneous applications [21, 69].

Despite the small number of studies, the observed 65% reduction in the risk of severe oral pain is noteworthy. A recent meta-analysis reported that ~ 31% of patients experience persisting oral pain after head and neck cancer treatment [24], which may result from neglected or inadequately managed oral pain during the initial treatment stages [8]. Our finding indicates that photobiomodulation may exert a prophylactic effect, preventing or attenuating the onset and progression of oral mucositis in patients undergoing radiotherapy. The underlying mechanisms of photobiomodulation may involve enhanced cell metabolism [35, 36], modulation of immune and inflammatory responses [3, 51], and the release of endogenous opioids [32, 38].

Although a statistically significant reduction was observed for severe oral pain (dichotomous outcome), no significant effects were found for continuous oral pain scores [2, 6, 15, 45, 55, 74]. This apparent discrepancy may reflect methodological and clinical differences between outcome measures. Dichotomous outcomes capture the occurrence of severe pain episodes, whereas continuous scales assess mean pain levels across the entire symptom spectrum. As observed, low baseline pain levels (< 3 points at baseline in 5 out of 6 studies [6, 15, 45, 55, 74]) may have limited the ability of continuous scales to detect meaningful between-group differences, particularly in the presence of floor effects [78]. In addition, substantial heterogeneity observed in the analysis (*I*^2^ = 98%) further limits interpretability. Therefore, the apparent analgesic effects of photobiomodulation should be interpreted cautiously, and further well-designed trials are needed to clarify its effects across different dimensions of pain and treatment.

The interplay between oral mucositis, xerostomia, and oral pain is commonly observed during the initial weeks of treatment [62] and can significantly impact the physical, psychological, and social well-being of patients with head and neck cancer [53]. We found that photobiomodulation appeared to stimulate salivary flow rates; however, its effects on xerostomia were trivial and did not reach statistical significance. The reasons for these may be related to the small number of studies included in the meta-analyses and variability of tools undertaken to assess xerostomia [43, 47, 70]. Nevertheless, such a finding suggests that photobiomodulation may help preserve salivary flow and at least prevent further exacerbation of xerostomia, hence reducing the risk of persistent oral mucositis, oral pain, dysphagia, infections, and osteoradionecrosis [1, 44].

We observed that photobiomodulation may help preserve quality of life, potentially reflecting some of the benefits observed across the outcomes of interest in this review. This finding suggests that managing symptoms and cancer treatment-related side effects in head and neck cancer through a patient-centered approach may help optimize supportive care and improve the overall treatment trajectory. Given that oral mucositis, xerostomia, and oral pain can significantly impair eating, speaking, and social interaction, counteracting these symptoms can enhance patients’ daily functioning and emotional well-being. However, substantial clinical and methodological heterogeneity posed challenges to interpretation, including variations in cancer stage, concomitant therapies, intervention timing, and quality-of-life instruments. Substantial statistical heterogeneity (*I*^2^ = 88%) was also observed. Although SMD were used to pool results, the diversity of settings and questionnaires limits clinical interpretability, and the overall effect should be interpreted cautiously as a broad indicator of benefit rather than a precise clinical estimate.

Strengths of this study are (i) the inclusion of 1653 patients with head and neck cancer across 28 randomized trials and (ii) a pairwise and subgroup meta-analysis exploring the effects of photobiomodulation and potential effect modifiers in this setting. Some limitations should also be acknowledged. First, a relatively large number of studies did not provide sufficient information on the effects of photobiomodulation on the outcomes of interest, which limits the precision and robustness of pooled estimates and precludes subgroup and dose–response analyses. Second, there was a variety of tools and criteria used to assess mucositis severity, xerostomia, oral pain, and quality of life across studies, increasing heterogeneity and potentially affecting the comparability of results and precision. Third, the meta-analyses comprised a relatively small number of studies, most of which presented high risk of bias. This raises substantial concerns regarding internal validity, and it is likely that the true magnitude of benefit may be lower than the pooled estimates indicate. For example, outcomes such as oral pain, xerostomia, and quality of life are highly subjective and particularly susceptible to placebo effects when participant and personnel blinding is lacking. Fourth, inadequate allocation concealment in many trials may have introduced selection bias, which can artificially inflate perceived benefits. For oral mucositis, an unblinded assessment can introduce detection bias, favouring the experimental group. Fifth, we also noted potential issues with selective reporting across the literature, where nonsignificant outcomes or less favorable time-points may have been omitted by original authors, further exaggerating the overall effect sizes. Greater transparency on randomization and allocation aspects and data reporting must be considered in future photobiomodulation trials to minimize bias and achieve a high certainty of evidence. Sixth, only the longest follow-up timepoint was analyzed for outcomes assessed at multiple timepoints, which may introduce attrition bias and limit interpretation of short-term effects. Finally, the small number of trials judged to be at low risk of bias for most outcomes precluded sensitivity analyses. Future updates including a greater number of well-conducted trials will be important to confirm the robustness of these findings.

In conclusion, this systematic review and meta-analysis suggests that photobiomodulation may reduce the risk of severe mucositis and oral pain, while potentially improving salivary flow rates and preserving quality of life in patients with head and neck cancer. However, the certainty of the evidence ranged from very low to low across outcomes, and most included studies presented a high risk of bias; therefore, these findings should be interpreted with caution. Overall, photobiomodulation appears to be a promising supportive care intervention with the potential to mitigate treatment-related toxicities and support quality of life. Future high-quality, standardized randomized controlled trials are required to optimize treatment parameters and strengthen the evidence base.

## Supplementary Information

Below is the link to the electronic supplementary material.

ESM 1 DOCX (473 KB)

## Data Availability

Data used in the present study such as data extraction templates, forms and analysis will be made available upon reasonable request to the corresponding author.
