# Supplementary material for: Effects of photobiomodulation on oral mucositis, oral pain, xerostomia, salivary flow rate, and quality of life in patients with head and neck cancer: a systematic review and meta-analysis
Source: Support Care Cancer. 2026 Mar 25;34(4):364. doi: 10.1007/s00520-026-10575-4 (PMC13018037; doi:10.1007/s00520-026-10575-4)
Supplement: Supplementary file 1 — DOCX (473 KB) [file 520_2026_10575_MOESM1_ESM.docx]

**Effects of photobiomodulation on oral mucositis, oral pain, xerostomia, salivary flow rate and quality of life in patients with head and neck cancer: A systematic review and meta-analysis**

**Study, participant, and intervention characteristics**

A total of 1,748 patients with head and neck cancer were included, of whom 872 were randomised to photobiomodulation. The majority of the sample were male (82.2%), with a median age of 57.7 (IQR: 55.6, 60.5) years. The most common cancer sites were the oropharynx (35.3%), followed by the oral cavity (26.6%) and hypopharynx (5.5%). Half of the patients had advanced disease (stage III-IV, n= 911, 55.1%) and 340 patients presented with metastasis.

All patients were undergoing radiotherapy, with total prescribed doses ranging from 50 to 70 Gy, delivered in 20 to 35 fractions of 1.8 to 2.0 Gy per session. In regard to chemotherapy, cisplatin-based protocols were the most common (18 [1, 2, 4, 6, 9-11, 14-16, 18, 20-23, 25-29, 33] out of 21 studies, 85.7%), administered either weekly (e.g., 40 mg.m^-2^) [15, 16] or at higher doses every three weeks (e.g., 100 mg.m^-2^) [1, 9-11, 28] in 66.7% of the patients. Some studies also had cisplatin combined with other agents such as 5-fluorouracil [4, 14, 18, 21, 22, 25-27], docetaxel [4, 18], cetuximab [14, 20], or carboplatin [14, 29].

Thirty-one photobiomodulation protocols were analysed. The protocols were undertaken during radiotherapy [1, 5-8, 12-16, 18, 19, 21-25, 27, 29, 32, 33] or ranging from 6-7 weeks [3, 4, 9-11, 20, 26, 28-30, 32, 34], except for one study [2] conducted over a single week and other conducted for 12 weeks [17]. Most studies applied photobiomodulation five times per week (51.6%) [1, 3, 5, 6, 8-11, 14, 15, 19, 21, 22, 26, 32, 34], while others used frequencies of two (16.1%) [7, 17, 29, 30, 33] or three times (19.4%) [4, 12, 18, 23, 25, 28] per week. Two studies [16, 20] did not report sufficient information about photobiomodulation frequency. All studies applied photobiomodulation once daily, except for one study [34] that applied it twice daily and two studies [16, 20] that did not report sufficient information regarding daily frequency.

Twenty-one (67.7%) studies used diode light source, including three studies that specifically used gallium aluminium arsenide (GaAIAs) devices, followed by five studies (16.1%) that used helium-neon (He-Ne) light source. One study reported the use of low-level polarized light therapy (RLPT). The emission spectrum was set between 630 and 660 nm in 13 studies (44.8%) and between 780 and 850 nm in 9 studies (27.6%). A few studies used emission spectrum outside these common ranges for superficial or deeper tissue penetration, including 600 nm [1], 685 nm [26, 27], 870 nm [24] and 940 nm [20]. Most studies (77.4%) used continuous emission mode, while two studies [14, 29] used pulsed emission mode. The average power output varied between 0.01 and 0.5 W, with beam spot size at the target area ranging from 0.03 to 1.00 cm^2^. The characteristic of studies is presented in **Supplementary** **Table S1**. Regarding the risk of bias, a high risk of bias was identified in 81.8% of studies assessing mucositis, 66.6% of studies assessing oral pain, 60.0% of studies assessing xerostomia and salivary flow rates, and 72.7% of studies assessing quality of life (**Supplementary** **Figures S2-S6**).

**Study risk of bias assessment and certainty of evidence (GRADE)**

The risk of bias was evaluated according to the 2^nd^ version of the Cochrane risk-of-bias tool for randomised trials (RoB 2) [31], with each assessment focused on the outcome level. The six-domain instrument includes: (1) randomisation process; (2) deviation from intended interventions; (3) missing outcome data; (4) measurement of the outcome; (5) selection of the reported result; and (6) overall bias. Overall risk of bias was expressed as “low risk of bias” if all domains were classified as low risk, “some concerns” if some concern was raised in at least one domain but not classified as at high risk in any other, or “high risk of bias” if at least one domain was classified as high risk, or two domains or more had some concerns [31]. The study risk of bias assessment for all included studies was performed independently by two reviewers (CTM and PC), with disagreements resolved by consensus.

The certainty of evidence was assessed using the *Grading of Recommendations Assessment, Development and Evaluation* (GRADE) approach. The several domains considered for downgrading the certainty of evidence were risk of bias, imprecision, inconsistency, indirectness and publication bias (i.e., based on comparison-adjusted funnel plot). The GRADE approach was not specified in the original PROSPERO protocol and was incorporated as a protocol amendment to enhance transparency through formal assessment of certainty of evidence. Its inclusion did not alter the pre-specified eligibility criteria, outcomes, or analytical methods.

**Appendix S1.** Search strategy.

**SEARCH STRATEGY**

**PubMed**

(“Head and Neck Cancer” [title/abstract] OR Head and Neck Neoplasm [title/abstract] OR Head, Neck Neoplasms [title/abstract] OR Neoplasms, Head and Neck [title/abstract] OR Head Neoplasms [title/abstract] OR Head Neoplasm [title/abstract] OR Neoplasm, Head [title/abstract] OR Neoplasms, Head [title/abstract] OR Neck Neoplasms [title/abstract] OR Neck Neoplasm [title/abstract] OR Neoplasm, Neck [title/abstract] OR Neoplasms, Neck [title/abstract] OR Cancer of Head and Neck [title/abstract] OR Cancer of the Head and Neck [title/abstract] OR Head and Neck Cancer [title/abstract] OR Upper Aerodigestive Tract Neoplasms [title/abstract] OR Upper Aerodigestive Tract Neoplasm [title/abstract] OR UADT Neoplasm [title/abstract] OR Neoplasms, UADT [title/abstract] OR Neoplasm, UADT [title/abstract] OR Neoplasms, Upper Aerodigestive Tract [title/abstract] OR UADT Neoplasms [title/abstract] OR Cancer of Neck [title/abstract] OR Neck Cancers [title/abstract] OR Cancer of the Neck [title/abstract] OR Neck Cancer [title/abstract] OR Cancer, Neck [title/abstract] OR Cancers, Neck [title/abstract] OR Cancer of Head [title/abstract] OR Head Cancers [title/abstract] OR Cancer of the Head [title/abstract] OR Head Cancer [title/abstract] OR Cancer, Head [title/abstract] OR Cancers, Head [title/abstract]) AND ("Laser Therapy" [title/abstract] OR "Low-Level Light Therapy" [title/abstract] OR "Phototherapy" [title/abstract] OR "Lasers" [title/abstract] OR "Photobiomodulation" [title/abstract])

**EBSCO host (CINAHL) + Web of Science**

(“Head and Neck Cancer” OR Head and Neck Neoplasm OR Head, Neck Neoplasms OR Neoplasms, Head and Neck OR Head Neoplasms OR Head Neoplasm OR Neoplasm, Head OR Neoplasms, Head OR Neck Neoplasms OR Neck Neoplasm OR Neoplasm, Neck OR Neoplasms, Neck OR Cancer of Head and Neck OR Cancer of the Head and Neck OR Head and Neck Cancer OR Upper Aerodigestive Tract Neoplasms OR Upper Aerodigestive Tract Neoplasm OR UADT Neoplasm OR Neoplasms, UADT OR Neoplasm, UADT OR Neoplasms, Upper Aerodigestive Tract OR UADT Neoplasms OR Cancer of Neck OR Neck Cancers OR Cancer of the Neck OR Neck Cancer OR Cancer, Neck OR Cancers, Neck OR Cancer of Head OR Head Cancers OR Cancer of the Head OR Head Cancer OR Cancer, Head OR Cancers, Head ) AND ("Laser Therapy" OR "Low-Level Light Therapy" OR "Phototherapy" OR "Lasers" OR "Photobiomodulation")

**Embase**

(((exp head/ and neck tumor/) or ((((((head and neck cancer) or head) and neck neoplasm*) or head) and neck tumor*) or HNC or "upper aerodigestive tract").ti,ab.) and (exp laser therapy/ or exp low level laser therapy/ or exp photobiomodulation/ or (photobiomodulation or "low level laser therapy" or "low level light therapy" or LLLT or laser*).ti,ab.)) not (exp animal/ not exp human/)

**LILACS and Scielo**

("Neoplasias de Cabeça e Pescoço" OR "Câncer de Cabeça e Pescoço" OR "Tumores de Cabeça e Pescoço" OR "Câncer Orofaringeano" OR "Câncer de Laringe" OR "Câncer Bucal" OR "Neoplasias de Cabeza y Cuello" OR "Cáncer de Cabeza y Cuello" OR "Tumores de Cabeza y Cuello" OR "Cáncer de Orofaringe" OR "Cáncer de Laringe" OR "Cáncer Oral") AND ("Terapia a Laser" OR "Terapia com Laser de Baixa Intensidade" OR "Laserterapia" OR "Fotobiomodulação" OR "Terapia Fotodinâmica" OR "Fototerapia" OR "Terapia con Láser" OR "Terapia Láser de Baja Intensidad" OR "Laserterapia" OR "Fotobiomodulación" OR "Terapia Fotodinámica" OR "Fototerapia")

**Table S1.** Participant, treatment and intervention characteristics of included studies.

| **Author** | **Participant characteristics** | **Treatment status** | **Intervention duration** | **Light source** | **Photobiomodulation parameters** | **Outcomes** |
| --- | --- | --- | --- | --- | --- | --- |
| Bensadoun  et al.  1999 | 30 participants with head and neck cancer  Age: Mean of 60.4 years  Males: 86.6%  Stage III-IV: NR | During radiotherapy: 100% | 7 weeks  1 session /  5 days a week | He–Ne | Emission spectrum (nm): 632.8  Mode: continuous  Average radiant power (W): 0.025 & 0.06  Beam spot size at target area (cm²): NR  Radiant exposure (J/cm²): 2  Points irradiated: NR  Time (s): 300 - 720 | Oral pain |
| Lopes  et al.  2006 | NR participants with head and neck cancer  Age: NR  Males:NR  Stage III-IV: NR | NR | NR | NR | NR | Salivary rate flow  Oral pain |
| Oton-Leite  et al.  2011 | 60 participants with head and neck cancer  Age: Median of 55.6 years  Males:81.6%  Stage III-IV: NR | During radiotherapy: 100% | 6 – 7 weeks  1 session /  5 days a week | Diode | Emission spectrum (nm): 685  Mode: continuous  Average radiant power (W): 0.035  Beam spot size at target area (cm²): NR  Radiant exposure (J/cm²): 2  Points irradiated: 37  Time (s): NR | Quality of life |
| Lima  et al.  2012 | 75 participants with head and neck cancer  Age: Median of 55 years  Males:76%  Stage III-IV: NR | During radiotherapy: 100%  During chemotherapy: 100% | During RT  1 session /  5 days a week | GaAIAs | Emission spectrum (nm): 660  Mode: NR  Average radiant power (W): 0.01  Beam spot size at target area (cm²): 0.4  Radiant exposure (J/cm²): 2  Points irradiated: NR  Time (s): NR | Oral mucositis |
| Gautam  et al.  2012 & Gautam  et al.  2012 & Gautam  et al.  2013 | 239 participants with head and neck cancer  Age: Mean of 55.5 years  Males: NR  Stage III-IV: 100% | During radiotherapy: 100%  During chemotherapy: 100% | 45 days  1 session /  5 days a week | He–Ne | Emission spectrum (nm): 632.8  Mode: continuous  Average radiant power (W): 0.024  Beam spot size at target area (cm²): 1  Radiant exposure (J/cm²): 3  Points irradiated: NR  Time (s): 145 | Oral mucositis  Oral pain  Quality of life |
| Antunes  et al.  2013 | 94 participants with head and neck cancer  Age: Mean of 54.6 years  Males:87.2%  Stage III-IV: 84% | During radiotherapy: 100%  During chemotherapy: 100% | During RT  1 session /  5 days a week | Diode | Emission spectrum (nm): 600  Mode: continuous  Average radiant power (W): 0.1  Beam spot size at target area (cm²): 0.24  Radiant exposure (J/cm²): 4  Points irradiated: NR  Time (s): 720 | Oral mucositis  Oral pain  Quality of life |
| Saleh  et al.  2014 | 23 participants with head and neck cancer  Age: Mean of 57.1 years  Males: 68%  Stage III-IV: 26% | Pre-radiotherapy: 100% | 6 weeks  1 session /  2 days a week | Diode | Emission spectrum (nm): 830  Mode: continuous  Average radiant power (W): 0.1  Beam spot size at target area (cm²): 0.028  Radiant exposure (J/cm²): 2  Points irradiated: 14  Time (s): NR | Xerostomia  Quality of life |
| Gautam  et al.  2015 | 49 participants with head and neck cancer  Age: Mean of 70.5 years  Males: 84%  Stage III-IV: 100% | During radiotherapy: 100% | During RT  1 session /  5 days a week | He–Ne | Emission spectrum (nm): 632.8  Mode: continuous  Average radiant power (W): 0.024  Beam spot size at target area (cm²): 1  Radiant exposure (J/cm²): 3  Points irradiated: 12  Time (s): NR | Oral mucositis  Oral pain |
| Oton-Leite  et al.  2015 | 30 participants with head and neck cancer  Age: NR  Males:84%  Stage III-IV: NR | Pre-surgery: 48%  During radiotherapy: 100%  During chemotherapy: 100% | 7 weeks  1 session /  3 days a week | Diode | Emission spectrum (nm): 660  Mode: continuous  Average radiant power (W): 0.025  Beam spot size at target area (cm²): 0.04  Radiant exposure (J/cm²): 6.2  Points irradiated: 69  Time (s): NR | Oral mucositis |
| Gonnelli  et al.  2016 | 23 participants with head and neck cancer  Age: NR  Males: 87%  Stage III-IV: 91.3% | Pre-surgery: 34.8%  During radiotherapy: 100%  During chemotherapy: 100% | During RT  1 session /  3 days a week | Diode | Emission spectrum (nm): 780 & 660  Mode: continuous  Average radiant power (W): 0.012 & 0.04  Beam spot size at target area (cm²): 0.04  Radiant exposure (J/cm²): 3.8 & 16.2  Points irradiated: 16 & 24  Time (s): NR | Salivary rate flow |
| Libik  et al.  2017 | 27 participants with head and neck cancer  Age: Median of 58.9 years  Males:75.9%  Stage III-IV: 100% | During radiotherapy: 100%  During chemotherapy: 100% | During RT  1 session /  5 days a week | He–Ne | Emission spectrum (nm): 630  Mode: continuous  Average radiant power (W): 0.03  Beam spot size at target area (cm²): NR  Radiant exposure (J/cm²): 5.16 & 16.2  Points irradiated: NR  Time (s): NR | Oral mucositis  Xerostomia  Salivary rate flow |
| Zhang  et al.  2018 | 60 participants with head and neck cancer  Age: Range of 24 to 75 years  Males: 70%  Stage III-IV: NR | During radiotherapy: 100% | 6 weeks  2 sessions /  5 days a week | RLPT | NR | Oral pain |
| Dantas  et al.  2019 | 54 participants with head and neck cancer  Age: Median of 56. 8 years  Males:83.9%  Stage III-IV: NR | During radiotherapy: 100%  During chemotherapy: 100% | 7 weeks  1 session /  3 days a week | Diode | Emission spectrum (nm): 660  Mode: continuous  Average radiant power (W): NR  Beam spot size at target area (cm²): 0.1256  Radiant exposure (J/cm²): 56  Points irradiated: 28  Time (s): 84 | Oral pain |
| Marin Conde  et al.  2019 | 36 participants with head and neck cancer  Age: Median of 60.8 years  Males:76.9%  Stage III-IV: 75% | During radiotherapy: 100%  During chemotherapy: 100% | NR | Diode | Emission spectrum (nm): 940  Mode: continuous  Average radiant power (W): 0.5  Beam spot size at target area (cm²): 0.36  Radiant exposure (J/cm²): NR  Points irradiated: 72  Time (s): 360 | Oral mucositis |
| Louzeiro  et al.  2020 | 27 participants with head and neck cancer  Age: Range of 18 to >70 years  Males:76.1%  Stage III-IV: 95.2 | Pre-surgery: 28.51%  During radiotherapy: 100%  Pre-chemotherapy: 38.9%  During chemotherapy: 61.9% | During RT  1 session /  3 days a week | Diode | Emission spectrum (nm): 660 & 810  Mode: continuous  Average radiant power (W): 0.4 & 4  Beam spot size at target area (cm²): 0.028  Radiant exposure (J/cm²): 10 & 25  Points irradiated: 22-80  Time (s): NR | Xerostomia  Salivary rate flow |
| Martins  et al.  2021 & Martins  et al.  2021 | 71 participants with head and neck cancer  Age: Mean of 59.75 years  Males:85.4%  Stage III-IV: NR | Pre-surgery: 45.8%  During radiotherapy: 100%  During chemotherapy: 89.6% | During RT  1 session /  5 days a week | Diode | Emission spectrum (nm): 660  Mode: continuous  Average radiant power (W): 0.025  Beam spot size at target area (cm²): 0.04  Radiant exposure (J/cm²): 6.2  Points irradiated: 61  Time (s): 720 | Oral mucositis  Quality of life |
| Robijns  et al.  2021 | 65 participants with head and neck cancer  Age: Mean of 64.39 years  Males:84.7%  Stage III-IV: NR | Pre-surgery: 54.34%  During radiotherapy: 100%  During chemotherapy:36.95% | 7 weeks  1 session /  5 days a week | Diode | Emission spectrum (nm): 808  Mode: pulsed  Average radiant power (W): 0.33  Beam spot size at target area (cm²): 3.14  Radiant exposure (J/cm²): 4  Points irradiated: NR  Time (s): NR | Quality of life |
| Kauark-Fontes et al.  2022 | 67 participants with head and neck cancer  Age: Mean of 60.7 years  Males:81.8%  Stage III-IV: 100% | Pre-surgery: 45.4%  During radiotherapy: 100% | During RT  1 session /  2 days a week | Diode | Emission spectrum (nm): 660  Mode: continuous  Average radiant power (W): 0.025  Beam spot size at target area (cm²): NR  Radiant exposure (J/cm²): 3  Points irradiated: NR  Time (s): NR | Oral mucositis  Quality of life |
| Mohamed  et al.  2022 | 100 participants with head and neck cancer  Age: Range of 18 to 80 years  Males:38.88%  Stage III-IV: 82.22% | Pre-surgery: 28.88%  During radiotherapy: 100%  During chemotherapy: 63.33% | During RT  1 session /  5 days a week | Diode | Emission spectrum (nm): 870  Mode: continuous  Average radiant power (W): 0.06  Beam spot size at target area (cm²): 0.55  Radiant exposure (J/cm²): 6  Points irradiated: 27  Time (s): NR | Oral mucositis  Oral pain |
| Sousa Melo  et al.  2022 | 36 participants with head and neck cancer  Age: Mean of 54.4 years  Males:77.8%  Stage III-IV: 42.05% | During radiotherapy: 100% | During RT  1 session /  3 days a week | GaAIAs | Emission spectrum (nm): 660  Mode: continuous  Average radiant power (W): 0.087  Beam spot size at target area (cm²): 0.03  Radiant exposure (J/cm²): 1  Points irradiated: 33  Time (s): NR | Oral mucositis |
| Carvalho e Silva et al.  2023 | 62 participants with head and neck cancer  Age: Mean of 60.5 years  Males:79.59%  Stage III-IV: NR | During radiotherapy: 100%  During chemotherapy: 100% | During RT  1 session /  5 days a week | Diode | Emission spectrum (nm): 660 & 808  Mode: continuous  Average radiant power (W): 0.1  Beam spot size at target area (cm²): 0.098  Radiant exposure (J/cm²): 0.3 & 1  Points irradiated: 221 & 22  Time (s): NR | Quality of life |
| Fernandes  et al.  2023 | 30 participants with head and neck cancer  Age: Mean of 58 years  Males:56.66%  Stage III-IV: NR | Pre-surgery: 23.3%  Pre-radiotherapy: 96.66%  Pre-chemotherapy: 96.66% | During RT  1 session /  2 days a week | Diode | Emission spectrum (nm): 660 & 780 & 808  Mode: continuous  Average radiant power (W): 0.04 & 0.07  Beam spot size at target area (cm²): NR  Radiant exposure (J/cm²): 2 & 3 & 9  Points irradiated: 3 & 9 & 10  Time (s): 40 & 50 | Quality of life |
| Mozzafari  et al.  2024 | 44 participants with head and neck cancer  Age: Mean of 55.6 years  Males: 29.72%  Stage III-IV: NR | During radiotherapy: 100%  During chemotherapy: 100% | During RT  1 session /  3 days a week | GaAIAs | Emission spectrum (nm): 810  Mode: continuous  Average radiant power (W): 0.2  Beam spot size at target area (cm²): 0.28  Radiant exposure (J/cm²): 2.85  Points irradiated: 16  Time (s): NR | Salivary rate flow |
| Barati  et al.  2025 | 36 participants with head and neck cancer  Age: Mean of 60.8 years  Males: 63.9%  Stage III-IV: NR | Pre-radiotherapy: 100%  Pre-chemotherapy: 100% | During RT | Diode | Emission spectrum (nm): 810  Mode: continuous  Average radiant power (W): 0.02  Beam spot size at target area (cm²): 1  Radiant exposure (J/cm²): 6  Points irradiated: NR  Time (s): NR | Oral mucositis  Oral pain  Quality of life |
| Camolesi et al. 2025 | 64 participants with head and neck cancer  Age: Mean of 64.3 years  Males: 67.9%  Stage III-IV: 86.7% | Pre-surgery: 20.7%  During radiotherapy: 30.2% | 6 weeks  1 session /  5 days a week | Diode | Emission spectrum (nm): 660 & 808  Mode: continuous  Average radiant power (W): 0.01  Beam spot size at target area (cm²): 0.03  Radiant exposure (J/cm²): NR  Points irradiated: 78  Time (s): 20 | Xerostomia  Salivary rate flow  Pain |
| Lopez-Garzon et al. 2025 | 31 participants with head and neck cancer  Age: Mean of 60.5 years  Males: 71.0%  Stage III-IV: 83.9% | Pre-surgery: 48.4%  Pre-radiotherapy: 100% | 12 weeks  1 session /  2 days a week | Diode | Emission spectrum (nm): 830  Mode: continuous  Average radiant power (W): 0.01  Beam spot size at target area (cm²): 0.02  Radiant exposure (J/cm²): 7.5  Points irradiated: 22  Time (s): 3 | Xerostomia  Salivary rate flow  Quality of life |

**
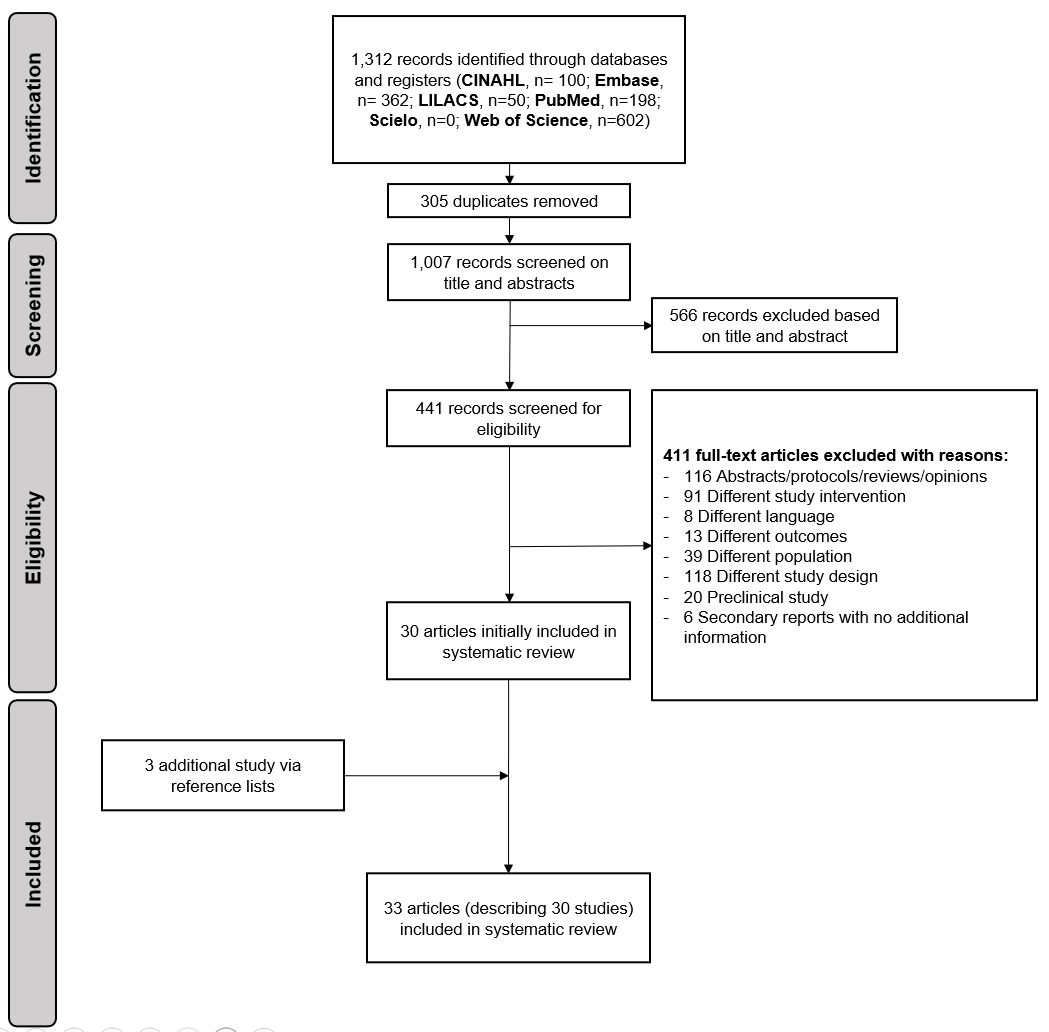
**

**Figure S1.** Flowchart of study selection.

**Figure S2.** Individual risk of bias assessment at outcome level for oral mucositis.

**Figure S3.** Individual risk of bias assessment at outcome level for oral pain.

**Figure S4.** Individual risk of bias assessment at outcome level for xerostomia.

**Figure S5.** Individual risk of bias assessment at outcome level for salivary flow rate.

**Figure S6.** Individual risk of bias assessment at outcome level for quality of life.

**
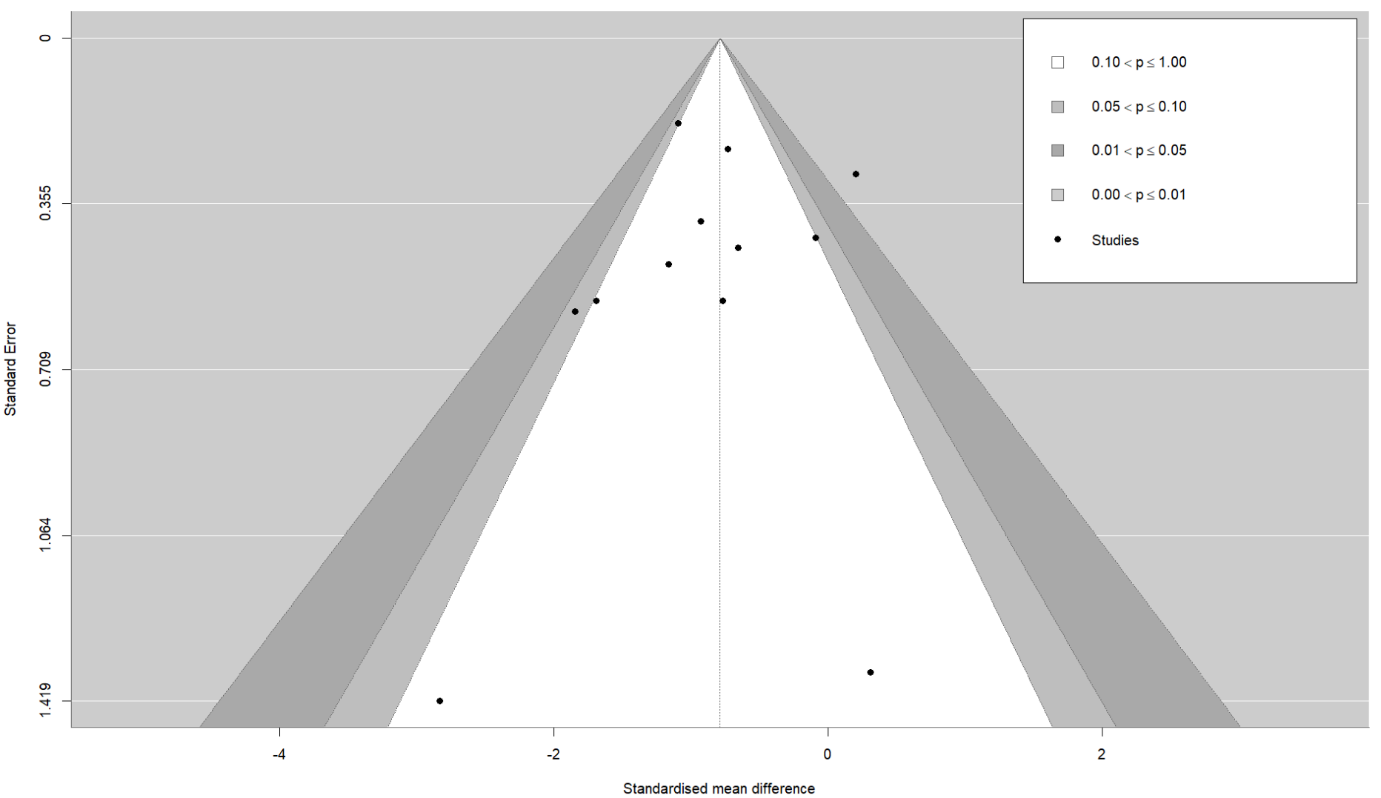
Figure S7**. Adjusted funnel plot for the effects of photobiomodulation on oral mucositis in patients with head and neck cancer.


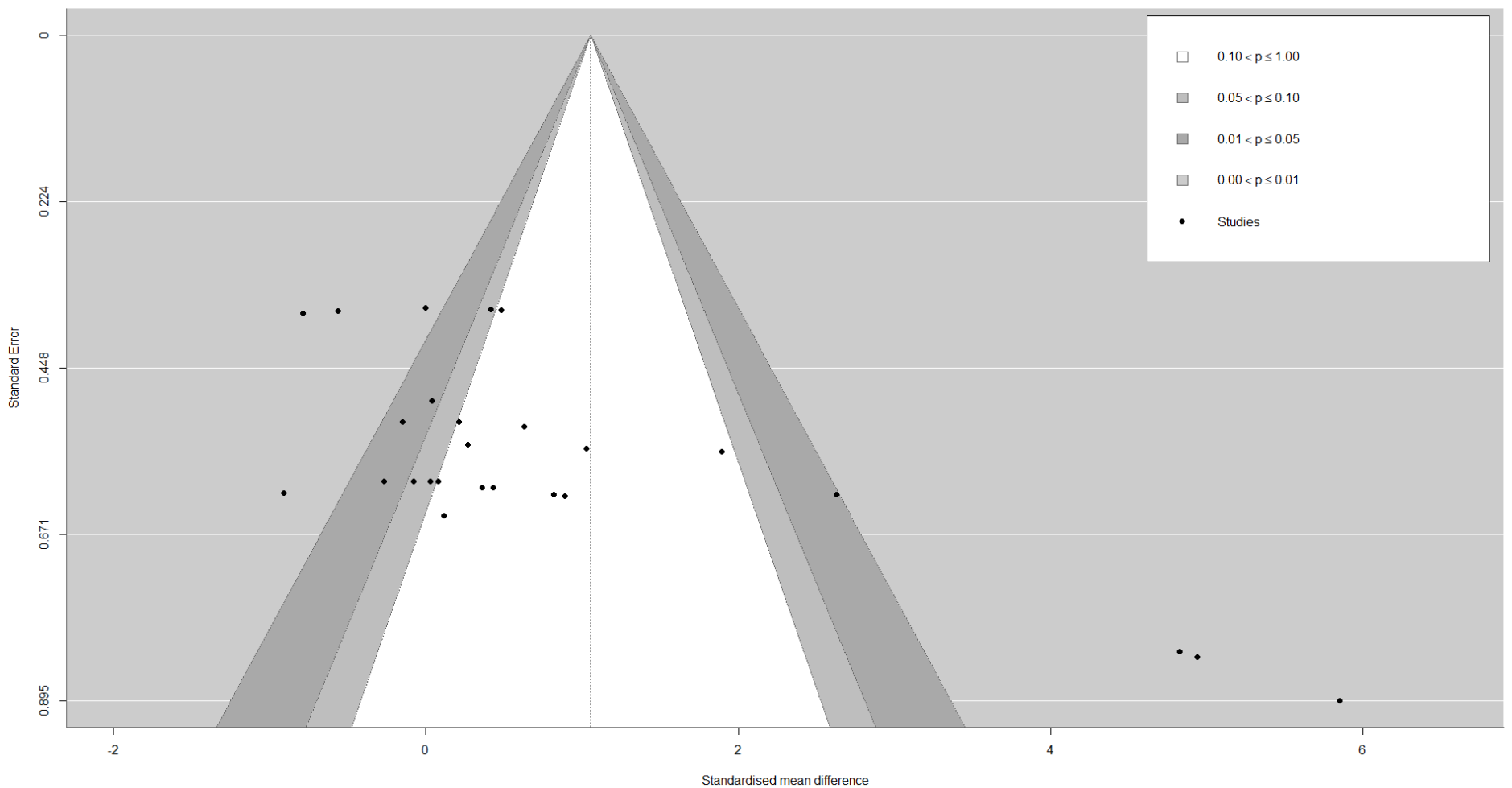
**Figure S8**. Adjusted funnel plot for the effects of photobiomodulation on quality of life in patients with head and neck cancer.

**Table S2.** Photobiomodulation effects on oral pain, xerostomia, salivary flow and quality of life in patients with head and neck cancer using data derived from correlation coefficients r = 0.25.

| **Outcomes** |  |  | **Random effect meta-analysis** | | | **Heterogeneity** | | |
| --- | --- | --- | --- | --- | --- | --- | --- | --- |
|  | **k** | **n** | **SMD** | **95% CI** | **P-value** | **Q** | **I^2^** | **P-value** |
| Oral pain | 6 | 6 | -2.25 | -5.47 to 0.96 | 0.170 | 98.9 | 98% | <0.001 |
| Xerostomia | 5 | 6 | -0.07 | -0.44 to 0.30 | 0.620 | 1.1 | 0% | 0.956 |
| Salivary flow | 6 | 10 | 0.62 | -0.02 to 1.27 | 0.055 | 9.8 | 26% | 0.365 |
| Quality of life | 11 | 27 | 1.06 | -0.03 to 2.14 | 0.060 | 145.2 | 88% | <0.001 |

95% CI, 95% confidence intervals; n, number of effect sizes; I^2^, percentage of variation across studies that is due to heterogeneity; k, number of studies; Q, Cochran’s Q test of heterogeneity.

**Table S3.** Photobiomodulation effects on oral pain, xerostomia, salivary flow and quality of life in patients with head and neck cancer using data derived from correlation coefficients r = 0.75.

| **Outcomes** |  |  | **Random effect meta-analysis** | | | **Heterogeneity** | | |
| --- | --- | --- | --- | --- | --- | --- | --- | --- |
|  | **k** | **n** | **SMD** | **95% CI** | **P-value** | **Q** | **I^2^** | **P-value** |
| Oral pain | 6 | 6 | -2.53 | -5.70 to 0.63 | 0.117 | 100.3 | 98% | <0.001 |
| Xerostomia | 5 | 6 | -0.14 | -0.59 to 0.31 | 0.430 | 1.7 | 0% | 0.895 |
| Salivary flow | 6 | 10 | 0.97 | 0.12 to 1.82 | 0.033 | 18.2 | 51% | 0.033 |
| Quality of life | 11 | 27 | 1.06 | -0.03 to 2.14 | 0.060 | 145.2 | 88% | <0.001 |

95% CI, 95% confidence intervals; n, number of effect sizes; I^2^, percentage of variation across studies that is due to heterogeneity; k, number of studies; Q, Cochran’s Q test of heterogeneity.

**REFERENCES**

1. Antunes HS, Herchenhorn D, Small IA, Araújo CM, Viégas CM, Cabral E, Rampini MP, Rodrigues PC, Silva TG, Ferreira EM, Dias FL, Ferreira CG (2013) Phase III trial of low-level laser therapy to prevent oral mucositis in head and neck cancer patients treated with concurrent chemoradiation Radiother Oncol 109: 297-302

2. Barati S, Motevasseli S, Saedi HS, Amiri P, Fekrazad R (2025) Effectiveness of Photobiomodulation (low-level laser therapy) on treatment of oral mucositis (OM) induced by chemoradiotherapy in head and neck cancer patients J Photochem Photobiol B 264: 113115

3. Bensadoun R, Franquin J, Ciais G, Darcourt V, Schubert M, Viot M, Dejou J, Tardieu C, Benezery K, Nguyen T (1999) Low-energy He/Ne laser in the prevention of radiation-induced mucositis: a multicenter phase III randomized study in patients with head and neck cancer Supportive care in cancer 7: 244-252

4. Dantas JBD, Martins GB, Lima HR, Carrera M, Reis SRD, Medrado A (2020) Evaluation of preventive laser photobiomodulation in patients with head and neck cancer undergoing radiochemotherapy: Laser in patients with head and neck cancer Special Care in Dentistry 40: 364-373

5. de Carvalho ESRM, Mendes FM, Degasperi GR, Pinheiro SL (2023) Photobiomodulation for the management of xerostomia and oral mucositis in patients with cancer: a randomized clinical trial Lasers Med Sci 38: 101

6. de Lima AG, Villar RC, de Castro G, Antequera R, Gil E, Rosalmeda MC, Federico MHH, Snitcovsky IML (2012) Oral mucositis prevention by low-level laser therapy in head-and-neck cancer patients undergoing concurrent chemoradiotherapy: A phase III randomized study International Journal of Radiation Oncology Biology Physics 82: 270-275

7. Fernandes AG, Oliveira AFd, Scarpel RD (2023) Fotobiomodulação no tratamento do trismo em pacientes tratados por câncer de boca ou orofaringe: um ensaio clínico controlado randomizado Audiol, Commun res 28: e2558-e2558

8. Gautam AP, Fernandes DJ, Vidyasagar MS, Maiya AG, Guddattu V (2015) Low level laser therapy against radiation induced oral mucositis in elderly head and neck cancer patients-a randomized placebo controlled trial J Photochem Photobiol B 144: 51-56

9. Gautam AP, Fernandes DJ, Vidyasagar MS, Maiya AG, Nigudgi S (2013) Effect of low-level laser therapy on patient reported measures of oral mucositis and quality of life in head and neck cancer patients receiving chemoradiotherapy--a randomized controlled trial Support Care Cancer 21: 1421-1428

10. Gautam AP, Fernandes DJ, Vidyasagar MS, Maiya AG, Vadhiraja BM (2012) Low level laser therapy for concurrent chemoradiotherapy induced oral mucositis in head and neck cancer patients - A triple blinded randomized controlled trial Radiotherapy and Oncology 104: 349-354

11. Gautam AP, Fernandes DJ, Vidyasagar MS, Maiya GA (2012) Low Level Helium Neon Laser therapy for chemoradiotherapy induced oral mucositis in oral cancer patients - A randomized controlled trial Oral Oncol 48: 893-897

12. Gonnelli FA, Palma LF, Giordani AJ, Deboni AL, Dias RS, Segreto RA, Segreto HR (2016) Low-Level Laser for Mitigation of Low Salivary Flow Rate in Head and Neck Cancer Patients Undergoing Radiochemotherapy: A Prospective Longitudinal Study Photomed Laser Surg 34: 326-330

13. Kauark-Fontes E, Migliorati CA, Epstein JB, Treister NS, Alves CGB, Faria KM, Palmier NR, Rodrigues-Oliveira L, de Pauli Paglioni M, Gueiros LAM, da Conceição Vasconcelos KGM, de Castro G, Jr., Leme AFP, Lopes MA, Prado-Ribeiro AC, Brandão TB, Santos-Silva AR (2022) Extraoral photobiomodulation for prevention of oral and oropharyngeal mucositis in head and neck cancer patients: interim analysis of a randomized, double-blind, clinical trial Support Care Cancer 30: 2225-2236

14. Legouté F, Bensadoun RJ, Seegers V, Pointreau Y, Caron D, Lang P, Prévost A, Martin L, Schick U, Morvant B, Capitain O, Calais G, Jadaud E (2019) Low-level laser therapy in treatment of chemoradiotherapy-induced mucositis in head and neck cancer: results of a randomised, triple blind, multicentre phase III trial Radiation Oncology 14

15. Libik TV, Gileva OS, Danilov KV, Grigorev SS, Pozdnyakova AA (2017) Management of Cancer Therapy-Induced Oral Mucositis Pain and Xerostomia with Extra- and Intra Oral Laser Irradiation. In: Editor (ed)^(eds) Book Management of Cancer Therapy-Induced Oral Mucositis Pain and Xerostomia with Extra- and Intra Oral Laser Irradiation, City.

16. Lopes CdO, Mas JRI, Zângaro RA (2006) Prevenção da xerostomia e da mucosite oral induzidas por radioterapia com uso do laser de baixa potência Radiol bras 39: 131-136

17. Lopez-Garzon M, Plata-Peregrina MDC, Perez-Sanchez EI, Lozano-Lozano M, Artacho-Cordon F, Galiano-Castillo N (2025) Photobiomodulation for restoring salivary flow after radiotherapy in head and neck cancer: a randomised placebo-controlled trial BMC Oral Health 25: 1468

18. Louzeiro GC, Cherubini K, de Figueiredo MAZ, Salum FG (2020) Effect of photobiomodulation on salivary flow and composition, xerostomia and quality of life of patients during head and neck radiotherapy in short term follow-up: A randomized controlled clinical trial Journal of Photochemistry and Photobiology B-Biology 209

19. Maiya GA, Sagar MS, Fernandes D (2006) Effect of low level helium-neon (He-Ne) laser therapy in the prevention & treatment of radiation induced mucositis in head & neck cancer patients Indian J Med Res 124: 399-402

20. Marín-Conde F, Castellanos-Cosano L, Pachón-Ibañez J, Serrera-Figallo MA, Gutiérrez-Pérez JL, Torres-Lagares D (2019) Photobiomodulation with low-level laser therapy reduces oral mucositis caused by head and neck radio-chemotherapy: prospective randomized controlled trial Int J Oral Maxillofac Surg 48: 917-923

21. Martins AFL, Morais MO, de Sousa-Neto SS, de Jesus APG, Nogueira TE, Valadares MC, Freitas NMA, Batista AC, Leles CR, Mendonça EF (2021) Photobiomodulation reduces the impact of radiotherapy on oral health-related quality of life due to mucositis-related symptoms in head and neck cancer patients Lasers Med Sci 36: 903-912

22. Martins AFL, Morais MO, Sousa-Neto SS, Oton-Leite AF, Pereira CH, Valadares MC, Freitas NMA, Batista AC, Leles CR, Mendonça EF (2021) The Effect of Photobiomodulation on Nitrite and Inflammatory Activity in Radiotherapy-Induced Oral Mucositis: A Randomized Clinical Trial Lasers Surg Med 53: 671-683

23. Melo AD, Andrade CL, Dantas JBD, Medrado A, Martins GB, Lima HR, Carrera M (2022) Impact of photobiomodulation for oral mucositis on body weight and BMI of patients with head and neck cancer Supportive Care in Cancer 30: 4897-4904

24. Mohamed NH, Kamel AM, Edress MF, Mahmoud ASS, Gaafar AIAE-H (2022) Low level laser therapy versus benzydamin in prevention and treatment of oral mucositis induced by anticancer treatments (clinical and biochemical study) Braz dent sci 25: 1-11

25. Mozaffari PM, Delavarian Z, Fekrazad R, Pakdel AF, Mohassel MR, Shakeri MT, Ghazi A (2024) Evaluation of the Effect of Photobiomodulation on Radiation-Induced Xerostomia in Head and Neck Cancer Patients: A Randomized Clinical Trial J Lasers Med Sci 15

26. Oton-Leite AF, Corrêa de Castro AC, Morais MO, Pinezi JC, Leles CR, Mendonça EF (2012) Effect of intraoral low-level laser therapy on quality of life of patients with head and neck cancer undergoing radiotherapy Head Neck 34: 398-404

27. Oton-Leite AF, Elias LS, Morais MO, Pinezi JC, Leles CR, Silva MA, Mendonça EF (2013) Effect of low level laser therapy in the reduction of oral complications in patients with cancer of the head and neck submitted to radiotherapy Spec Care Dentist 33: 294-300

28. Oton-Leite AF, Silva GB, Morais MO, Silva TA, Leles CR, Valadares MC, Pinezi JC, Batista AC, Mendonça EF (2015) Effect of low-level laser therapy on chemoradiotherapy-induced oral mucositis and salivary inflammatory mediators in head and neck cancer patients Lasers Surg Med 47: 296-305

29. Robijns J, Lodewijckx J, Claes S, Van Bever L, Pannekoeke L, Censabella S, Bussé L, Colson D, Kaminski I, Broux V, Puts S, Vanmechelen S, Timmermans A, Noé L, Bulens P, Govers M, Maes A, Mebis J (2021) Photobiomodulation therapy for the prevention of acute radiation dermatitis in head and neck cancer patients (DERMISHEAD trial) Radiother Oncol 158: 268-275

30. Saleh J, Figueiredo MAZ, Cherubini K, Braga A, Salum FG (2014) Effect of Low-Level Laser Therapy on Radiotherapy-Induced Hyposalivation and Xerostomia: A Pilot Study Photomedicine and Laser Surgery 32: 546-552

31. Sterne JAC, Savović J, Page MJ, Elbers RG, Blencowe NS, Boutron I, Cates CJ, Cheng HY, Corbett MS, Eldridge SM, Emberson JR, Hernán MA, Hopewell S, Hróbjartsson A, Junqueira DR, Jüni P, Kirkham JJ, Lasserson T, Li T, McAleenan A, Reeves BC, Shepperd S, Shrier I, Stewart LA, Tilling K, White IR, Whiting PF, Higgins JPT (2019) RoB 2: a revised tool for assessing risk of bias in randomised trials Bmj 366: l4898

32. Vianna Camolesi GC, Prado-Pena IB, Gómez-Caamaño A, Victoria-Fernández C, Blanco-Carrión A, García-García A, Gándara-Vila P, Pérez-Sayáns M (2025) Photobiomodulation for the prevention of oral side effects secondary to head and neck cancer therapy: results of a randomised, single-blind clinical trial Oral Oncol 164: 107266

33. Zanin T, Zanin F, Carvalhosa AA, Castro PH, Pacheco MT, Zanin IC, Brugnera A, Jr. (2010) Use of 660-nm diode laser in the prevention and treatment of human oral mucositis induced by radiotherapy and chemotherapy Photomed Laser Surg 28: 233-237

34. Zhang X, Li H, Li Q, Li Y, Li C, Zhu M, Zhao B, Li G (2018) Application of red light phototherapy in the treatment of radioactive dermatitis in patients with head and neck cancer World J Surg Oncol 16: 222
